# Comparison of the Efficacy and Safety of Minimally Invasive Simple Prostatectomy and Endoscopic Enucleation of Prostate for Large Benign Prostatic Hyperplasia

**DOI:** 10.3389/fmed.2021.773257

**Published:** 2021-11-05

**Authors:** Jinze Li, Dehong Cao, Chunyang Meng, Zhongyou Xia, Lei Peng, Yunxiang Li, Qiang Wei

**Affiliations:** ^1^Department of Urology/Institute of Urology, West China Hospital, Sichuan University, Chengdu, China; ^2^West China School of Clinical Medicine, Sichuan University, Chengdu, China; ^3^Department of Urology, Nanchong Central Hospital, The Second Clinical Medical College, North Sichuan Medical College, Nanchong, China; ^4^Department of Urology, The First Affiliated Hospital of Kunming Medical University, Kunming, China

**Keywords:** minimally invasive simple prostatectomy, endoscopic enucleation, benign prostatic hyperplasia, outcomes, meta-analysis

## Abstract

**Background:** Minimally invasive simple prostatectomy (MISP) and endoscopic enucleation of the prostate (EEP) are the two most commonly used methods for large benign prostatic hyperplasia (BPH), but it remains unclear which of the two is superior. This study aims to perform a pooled analysis to compare efficacy and safety profiles between MISP and EEP.

**Methods:** We conducted a comprehensive search of PubMed, Embase, Web of Science, and ClinicalTrials.gov databases to identify eligible studies comparing MISP with EEP. Parameters including efficacy and safety outcomes were compared using Stata 14.0 version.

**Results:** Eight comparative trials with 1,504 patients were included. Compared to MISP, EEP demonstrated shorter operative time (mean difference [MD] 46.37, 95% confidence interval [CI] 19.92 to 72.82, *p* = 0.0006), lesser hemoglobin decrease (standardized MD [SMD] 0.59, 95% CI 0.23 to 0.95, *p* = 0.001), lower catheterization time (SMD 4.13, 95% CI 2.16 to 6.10, *p* < 0.001), and shorter length of stay (SMD 2.38, 95% CI 1.40 to 3.36, *p* < 0.001). However, overall complications and blood transfusions did not differ between the two groups. Moreover, EEP had better postvoid residual volume (PVR) at 6-month (MD 14.39, 95% CI 11.06 to 17.72, *p* < 0.001) and comparable 3- and 6-month International Prostate Symptom Score, 3- and 6-month maximum flow rate, 3-month PVR, and 3-month quality of life compared with MISP.

**Conclusion:** Both MISP and EEP are effective and safe surgical procedures for the treatment of large BPH. EEP appears to have a superior perioperative profile compared to MISP. This should be interpreted with caution due to the significant heterogeneity between studies. Hence, treatment selection should be based on the surgeon's experience and availability.

## Introduction

Benign prostatic hyperplasia (BPH) is a common disease in elderly men, and its incidence increases gradually with age. The obstruction of the lower urinary tract caused by an enlarged prostate brings great trouble to the life of patients (1). Surgical intervention is required when patients with BPH have severe complications or non-responding well to pharmacological treatment (2). Current guidelines recommend open simple prostatectomy (OSP) as the gold standard procedure for large BPH (prostate volume > 80 mL) (3, 4), but it is related to higher postoperative complications and longer length of stay (LOS) (5, 6). For these reasons, several surgical approaches including endoscopic enucleation of the prostate (EEP) and minimally invasive simple prostatectomy (MISP) have been mentioned and introduced to reduce the morbidity associated with open surgery.

EEP based on laser or bipolar technology has proven to be a safe and efficacious treatment for BPH. A growing body of literature suggests that it has superior perioperative outcomes while providing the same clinical efficacy as OSP (7). Therefore, EEP is considered the standard therapy for treating large prostates (3). In recent years, laparoscopic and robotic techniques have been widely adopted in urological surgery because of their advantages of low trauma and quick recovery. MISP is a relatively new technique for large BPH through a laparoscopic or robot-assisted approach. This technique appears to be more attractive than OSP, as it offers less blood loss, shorter LOS, and lower perioperative complications (8). Both MISP and EEP are considered alternatives to OSP (7, 8), however, there is no common consensus as to which approach is most appropriate.

Therefore, this study aimed to incorporate available clinical studies to systematically compare the efficacy and safety of MISP and EEP for large BPH, and provide the latest evidence for clinical practice.

## Methods

This present study has been reported following the PRISMA (Preferred Reporting Items for Systematic Reviews and Meta-Analysis) statement (9) and has been registered on PROSPERO: CRD42021239950.

### Search Strategy

We conducted a comprehensive search of PubMed, Embase, Web of Science, and ClinicalTrials.gov databases to identify eligible studies comparing MISP and EEP for the treatment of large prostates and published in English from database establishment through August 2021. The database was searched using the following terms: “minimally invasive”, “laparoscopic”, “laparoscopy”, “robot”, “robotic”, “robot-assisted”, “robotic-assisted” “simple prostatectomy”, “adenomectomy”, “enucleation”, “prostate”, and “benign prostatic hyperplasia”. Moreover, relevant references for all eligible studies were also manually searched. Two authors independently reviewed the literature and any discrepancies were addressed through discussion with the third author.

### Identification of Eligible Studies

The inclusion criteria for the study were defined as follows: (1) comparative studies comparing one of the minimally invasive methods (laparoscopic simple prostatectomy [LSP] and robot-assisted simple prostatectomy [RASP]) with the EEP procedure (laser or bipolar enucleation) for the treatment of patients with prostate volume > 80 mL; (2) data on efficacy or safety outcomes (or both) were provided. Non-comparative studies, reviews, case reports, letters, meeting abstracts, unpublished studies, and non-English articles were excluded.

### Data Extraction

Data extraction was carried out independently by the two authors, and differences were resolved through discussion with the third author. The following data were extracted from the included studies: first author, publication year, country of study, study design, intervention, number of patients, age, body mass index (BMI), follow-up time, prostate volume, International Prostate Symptom Score (IPSS), maximum flow rate (Qmax), postvoid residual volume (PVR) quality of life (QoL), prostate-specific antigen (PSA), operative time, hemoglobin (Hb) decrease, catheterization time, LOS, resection weight, and complications. In addition, the data in the original study were converted to mean and standard deviation (SD) by computational expressions (10). If available, complications based on the Clavien-Dindo classification (CDC) system classification were analyzed.

### Quality Assessment

The quality of all included studies was assessed using the Newcastle-Ottawa scale (maximum score 9) (11). In the current meta-analysis, studies with scores ≥ 7 were considered high quality. Two independent reviewers evaluated the quality of included studies, and any differences were resolved through consultation with a third reviewer.

### Statistical Analysis

In this study, Stata 14.0 version (Stata Corp, College Station, TX, US) was used for statistical analysis. The mean difference (MD) or the standardized MD (SMD) and odds ratio (OR) were used as effect analysis statistics for continuous and dichotomous variables, respectively. The random-effects model was adopted in the analyses. Heterogeneity among studies was measured using the Chi-square (χ^2^) and inconsistency index (*I*^2^) tests, and *P* < 0.10 or *I*^2^ > 50% was regarded as significant heterogeneity. 95% confidence intervals (CIs) were calculated for all statistics. *P* < 0.05 was considered statistically significant. Furthermore, the sensitivity analysis was performed by excluding one or more studies that might have contributed to heterogeneity. Funnel plot and Begg's test were used to assess potential publication bias.

## Results

### Literature Search and Study Characteristics

**Figure 1:**
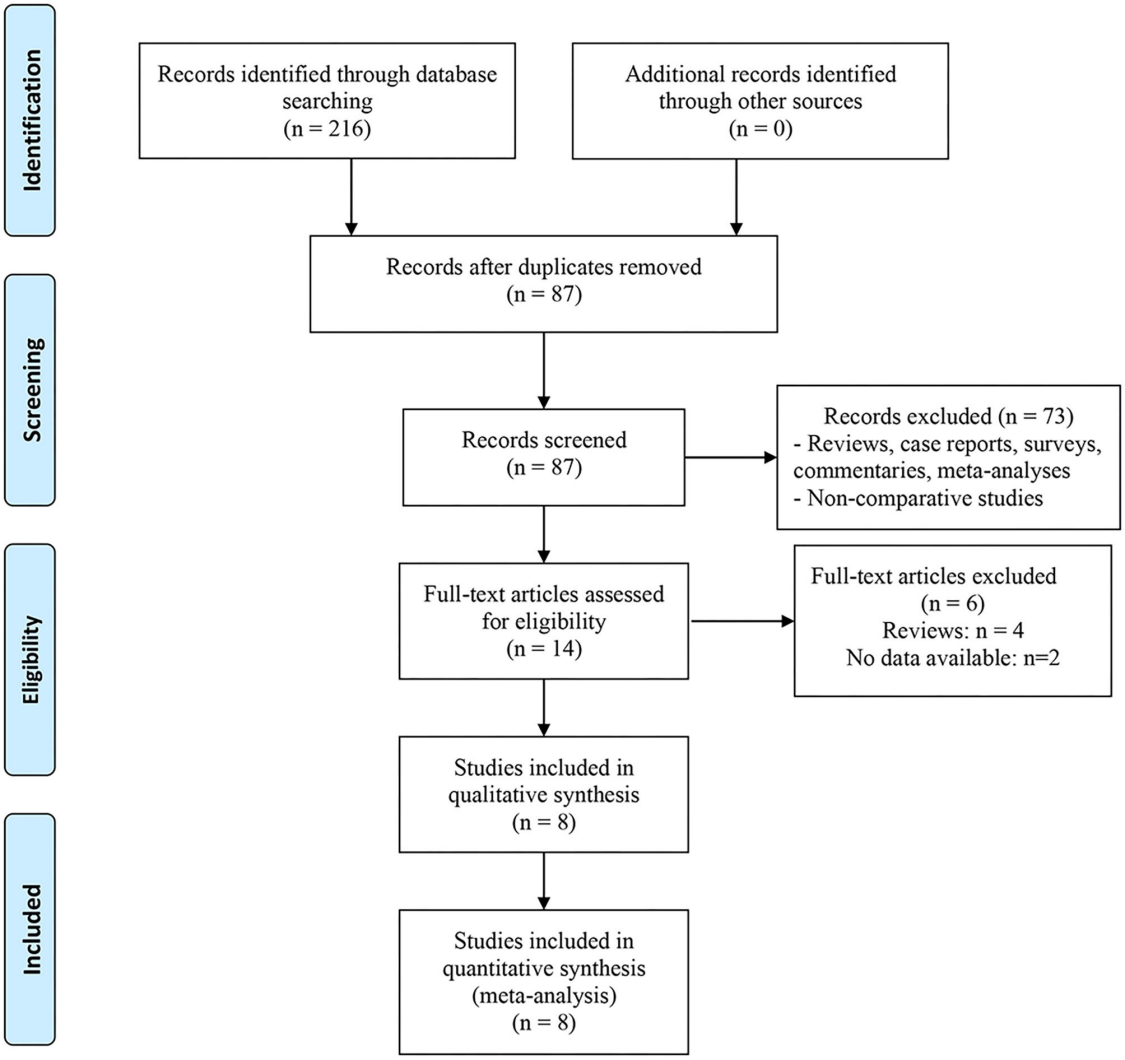
Flow diagram of for study selection.

The screening process is presented in Figure 1, 216 studies were preliminarily retrieved, and 87 remained after duplicates were removed. We excluded 73 studies after reviewing abstracts and titles, and 6 articles after reading the full text. Finally, 8 studies involving 1,504 patients (492 MISP vs. 1,012 EEP) were included in the present meta-analysis (12–19). These studies were published between 2015 and 2021. Among them, one was a prospective study (16), six were retrospective studies (12–15, 18, 19), and one was a prospective randomized study (17). Four studies (12, 13, 18, 19) focused on the comparison of LSP and EEP, three studies (14–16) focused on the comparison of RASP and EEP, and only one study (17) analyzed the comparison of LSP, RASP, and EEP. Additionally, since Fuschi et al. (17) reported the comparison of two MISP technologies (LSP and RASP) with EEP, we divided their results into two parts and presented them as Fuschi 2020a (LSP vs. EEP) and Fuschi 2020b (RASP vs. EEP) during data analysis. The characteristics of all patients in each study are presented in Tables 1, 2. The quality evaluation results indicated that all the studies were rated as high quality, and detailed scores for each study are shown in Supplementary Material 1.

**Table 1:** Characteristics and designs of the included studies.

**References**	**Country**	**Study design**	**Intervention**	**Patients (n)**	**Age (years)**	**BMI (kg/m^**2**^)**	**Follow-up (months)**	**Quality score^†^**
Lusuardi et al. (12)	Austria	Retrospective	MISP (laparoscopic) EEP (eraser laser)	20 20	74.7 ± 5.9 68.9 ± 6.7	NA	6	7
Baldini et al. (13)	France	Retrospective	MISP (laparoscopic) EEP (holmium laser)	28 39	68.6 ± 1.4 69.8 ± 1.3	NA	3	8
Umari et al. (14)	Italy	Retrospective	MISP (robot-assisted) EEP (holmium laser)	81 45	66.0 ± 7.4 74.0 ± 8.9	27 ± 5.2 26 ± 3.7	NA	8
Zhang et al. (15)	America	Retrospective	MISP (robot-assisted) EEP (holmium laser)	32 600	71.0 ± 8.0 71.0 ± 8.0	NA	2	7
Nestler et al. (16)	Germany	Prospective	MISP (robot-assisted) EEP (thulium laser)	35 35	70.9 ± 6.1 71.2 ± 7.2	NA	12	8
Fuschi et al. (17)	Italy	Prospective	MISP (laparoscopic) MISP (robot-assisted) EEP (holmium laser)	36 32 42	64.3 ± 7.1 69.4 ± 6.2 68.2 ± 6.1	21.8 ± 3.0 20.3 ± 3.1 23.8 ± 3.3	24	9
Gunseren et al. (18)	Turkey	Retrospective	MISP (laparoscopic) EEP (holmium laser)	61 60	70.2 ± 7.7 70.1 ± 7.5	25.4 ± 3.6 25.3 ± 2.0	3	7
Lombardo et al. (19)	France	Retrospective	MISP (laparoscopic) EEP (bipolar)	167 129	69.1 ± 7.8 73.1 ± 7.5	27.8 ± 2.8 28.1 ± 2.6	36	8

*MISP, minimally invasive simple prostatectomy; EEP, endoscopic enucleation of the prostate; BMI, body mass index; NA, not available; X±Y, mean±standard deviation;*

† *NOS, Newcastle-Ottawa scale*.

**Table 2:** Baseline characteristics of patients.

**References**	**Intervention**	**PV (mL)**	**PSA (ng/mL)**	**IPSS**	**QoL**	**Qmax (mL/s)**	**PVR (mL)**
Lusuardi et al. (12)	LSP ELEP	94.0 ± 22.4 96.1 ± 35.9	7.5 ± 3.3 8.1 ± 3.7	27.7 ± 5.0 28.4 ± 5.0	NA	7.8 ± 2.3 6.7 ± 2.6	142.5 ± 69.6 173.7 ± 82.5
Baldini et al. (13)	LSP HoLEP	120.5 ± 37.2 83.9 ± 28.8	8.4 ± 1.5 7.2 ± 0.7	19.8 ± 2.6 21.1 ± 1.0	4.0 ± 0.1 4.4 ± 0.2	7.5 ± 0.9 8.2 ± 0.8	159.4 ± 8.9 137.1 ± 3.0
Umari et al. (14)	RASP HoLEP	130.0 ± 58.5 130.0 ± 27.4	7.1 ± 6.1 8.6 ± 8.4	25.0 ± 5.9 21.0 ± 6.7	NA	8.0 ± 4.4 9.0 ± 5.2	73.0 ± 43.0 100.0 ± 95.6
Zhang et al. (15)	RASP HoLEP	>80	NA	24.0 ± 4.0 20.0 ± 7.0	NA	NA	NA
Nestler et al. (16)	RASP ThuLEP	94.5 ± 40.0 90.8 ± 35.6	NA	23.0 ± 3.7 20.0 ± 3.7	5.0 ±1.5 4.2 ± 1.5	NA	NA
Fuschi et al. (17)	LSP RASP HoLEP	143.8 ± 31.3 149.4 ± 35.2 142.2 ± 30.1	5.6 ± 3.5 5.2 ± 2.9 5.6 ± 3.3	23.4 ± 2.8 24.3 ± 1.9 24.2 ± 3.0	3.9 ± 0.8 3.8± 0.7 3.9 ± 0.8	7.1 ± 1.7 7.2 ± 2.3 7.1 ± 1.9	132.3 ± 31.3 126.1 ± 22.2 130.1 ± 33.5
Gunseren et al. (18)	LSP HoLEP	103.5 ± 23.3 99.5 ± 21.3	NA	NA	NA	NA	NA
Lombardo et al. (19)	LSP B-TUEP	108.1 ± 39.0 94.8 ± 15.0	7.9 ± 5.6 6.3 ± 3.4	21.0 ± 3.5 23.2 ± 5.7	2.1 ± 0.5 2.4 ± 0.7	9.5 ± 4.2 7.1 ± 3.8	85.0 ± 26.0 85.3 ± 26.1

*LSP, laparoscopic simple prostatectomy; RASP, robotic-assisted simple prostatectomy; ELEP, eraser laser enucleation of the prostate; HoLEP, holmium laser enucleation of the prostate; ThuLEP, thulium laser enucleation of the prostate; B-TUEP, bipolar transurethral enucleation of the prostate; PV, prostate volume; PSA, prostate specific antigen; IPSS, International Prostate Symptom Score; QoL, quality of life; Qmax, maximum flow rate; PVR, postvoid residual volume; NA, not available; X±Y, mean±standard deviation*.

### Efficacy Outcomes

**Figure 2:**
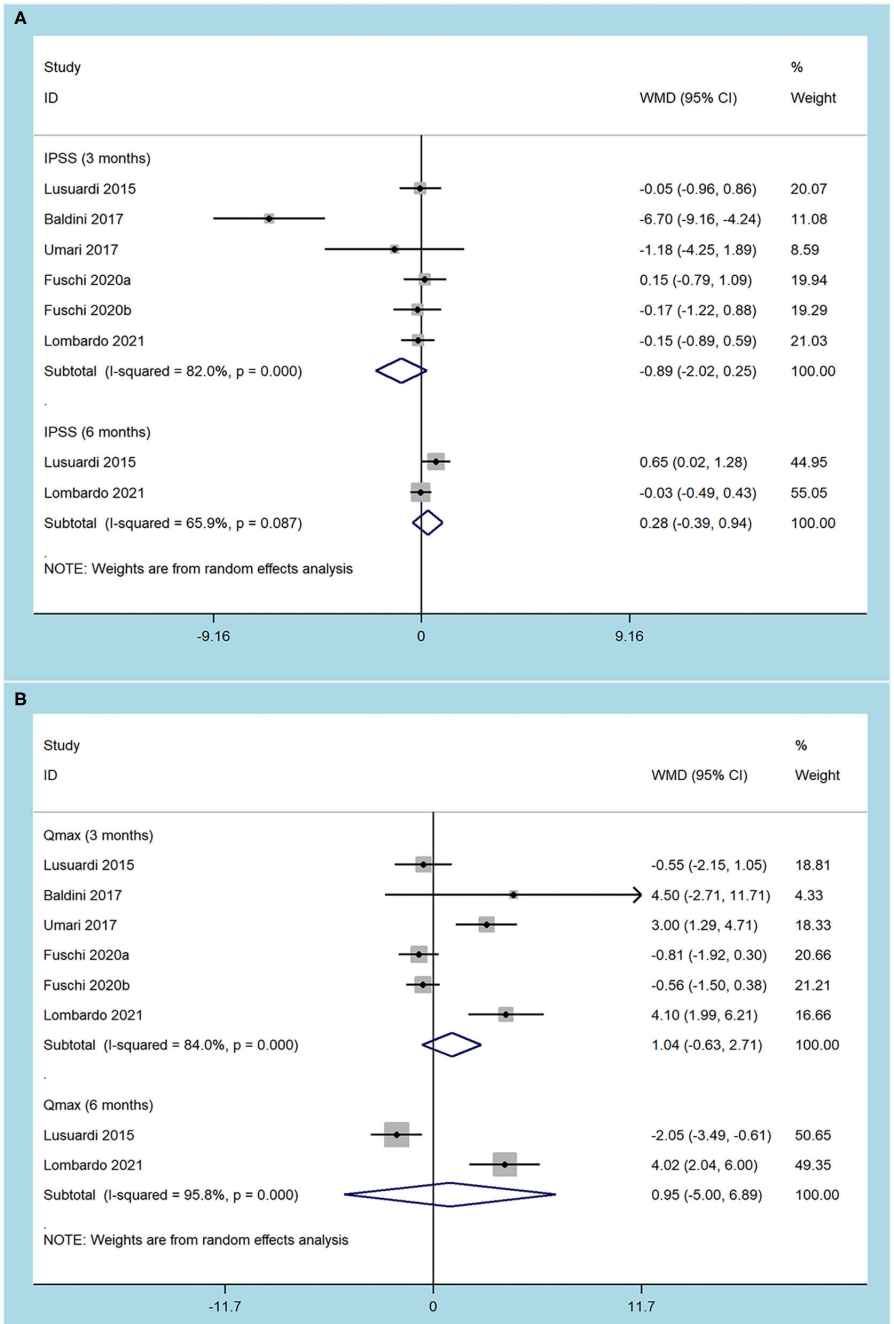
Forest plots of efficacy outcomes. **(A)** International prostate symptom score; **(B)** maximum flow rate.

**Figure 3:**
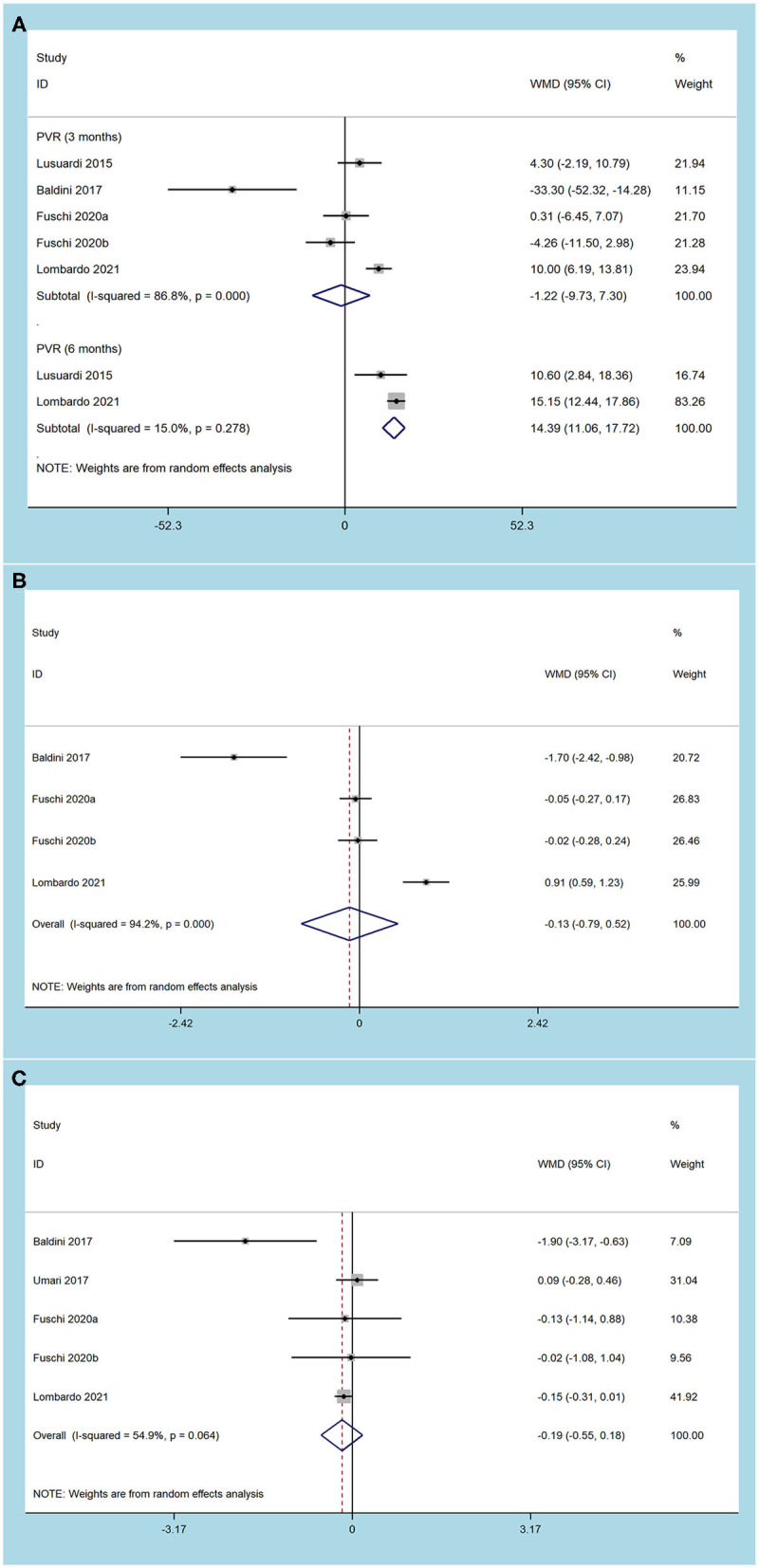
Forest plots of efficacy outcomes. **(A)** postvoid residual volume; **(B)** quality of life; **(C)** prostate specific antigen).

The short-term efficacy outcomes of MISP and EEP were evaluated in the meta-analysis. Five studies (12–14, 17, 19) showed that there were no significant differences in 3-month (MD −0.89, 95% CI −2.02 to 0.25, *p* = 0.13) and 6-month (MD 0.28, 95% CI −0.39 to 0.94, *p* = 0.42) IPSS between MISP and EEP groups (Figure 2). Five trials (12–14, 17, 19) reported postoperative Qmax data (Figure 2), and no significant differences were observed regarding Qmax between groups at 3-month (MD 1.04, 95% CI −0.63 to 2.71, *p* = 0.22) and 6-month (MD 0.95, 95% CI −5.00 to 6.89, *p* = 0.76). Postoperative PVR was obtained from 4 studies (12, 13, 17, 19) (Figure 3). The 3-month (MD−1.22, 95% CI −9.73 to 7.30, *p* = 0.78) PVR was similar between the groups. However, the MISP group had higher 6-month PVR (MD 14.39, 95% CI 11.06 to 17.72, *p* < 0.001) than the EEP group. Three trials (13, 17, 19) compared postoperative QOL in the MISP and EEP groups (Figure 3). There was no difference in 3-month QoL (MD −0.13, 95% CI −0.79 to 0.52, *p* = 0.69). Furthermore, four studies (13, 14, 17, 19) reported the postoperative PSA (Figure 3), and MISP had the same PSA as the EEP group during short-term follow-up (MD −0.19, 95% CI −0.55 to 0.18, *p* = 0.33). Of note, there was significant heterogeneity among studies (*p* < 0.10) for each metric.

### Perioperative Outcomes

The current analysis suggested that EEP had a shorter operative time (MD 46.37, 95% CI 19.92 to 72.82, *p* = 0.0006), lower Hb decrease (SMD 0.59, 95% CI 0.23 to 0.95, *p* = 0.001), shorter catheterization time (SMD 4.13, 95% CI 2.16 to 6.10, *p* < 0.001) and lower LOS (SMD 2.38, 95% CI 1.40 to 3.36, *p* < 0.001) compared to MISP. However, no difference was observed in the resection weight (MD 6.34, 95% CI −3.51 to 16.19, *p* = 0.21) between the groups. The heterogeneity test indicated that there was significant heterogeneity among studies of each outcome (*p* < 0.10) (Table 3).

**Table 3:** Subgroup analysis of perioperative outcomes and complications comparing MISP and EEP.

**Outcome**	**Variable**	**No. of studies**	**Patients (MISP/EEP)**	**MD/OR (95% CI)**	***P***	**Heterogeneity**	
						**I^**2**^**	***P***
**Operative time**	All	8	492/1,012	46.37 (19.92, 72.82)	0.0006	98%	<0.001
Minimally invasive approach	LSP RASP	5 4	312/290 180/722	29.19 (7.30, 51.08) 68.97 (−7.33, 145.27)	0.009 0.08	97% 99%	<0.001 <0.001
Hb decrease^*^	All	7	431/905	0.59 (0.23, 0.95)	0.001	83%	<0.001
Minimally invasive approach	LSP RASP	5 4	251/230 180/675	0.71 (−0.01, 1.49) 0.48 (0.20, 0.76)	0.05 0.0007	91% 45%	<0.001 0.14
Catheterization time^*^	All	8	492/1,002	4.13 (2.16, 6.10)	<0.001	99%	<0.001
Minimally invasive approach	LSP RASP	5 4	312/290 180/712	2.29 (1.60, 4.98) 5.11 (0.30, 9.92)	0.0001 0.04	98% 100%	<0.001 <0.001
LOS^*^	All	8	492/1011	2.38 (1.40, 3.36)	<0.001	97%	<0.001
Minimally invasive approach	LSP RASP	5 4	312/290 180/721	2.67 (1.11, 4.23) 2.11 (0.55, 3.68)	0.0008 0.008	98% 97%	<0.001 <0.001
**Resection weight**	All	7	430/952	6.34 (−3.51, 16.19)	0.21	86%	<0.001
Minimally invasive approach	LSP RASP	4 4	251/230 179/722	13.55 (−0.25, 27.34) −1.25 (−18.86, 16.35)	0.05 0.89	87% 85%	0.0001 <0.001
**Overall complications**	All	7	460/412	1.10 (0.76, 1.59)	0.62	6%	0.39
Minimally invasive approach	LSP RASP	5 3	308/290 152/122	0.99 (0.65, 1.51) 1.71 (0.60, 4.93)	0.97 0.31	0% 55%	0.69 0.11
**Blood transfusions**	All	5	323/863	1.83 (0.66, 5.08)	0.24	45%	0.12
Minimally invasive approach	LSP RASP	3 2	256/228 67/635	0.95 (0.46, 1.96) 5.84 (1.73, 19.70)	0.89 0.004	0% 0%	0.68 0.83

*MISP, minimally invasive simple prostatectomy; EEP, endoscopic enucleation of the prostate; LSP, laparoscopic simple prostatectomy; RASP, robotic-assisted simple prostatectomy; HoLEP, holmium laser enucleation of the prostate; LOS, length of stay; MD, mean difference; CI, confidence interval*.

* *Indicates the use of standardized MD as the effect statistic*.

### Complications

**Figure 4:**
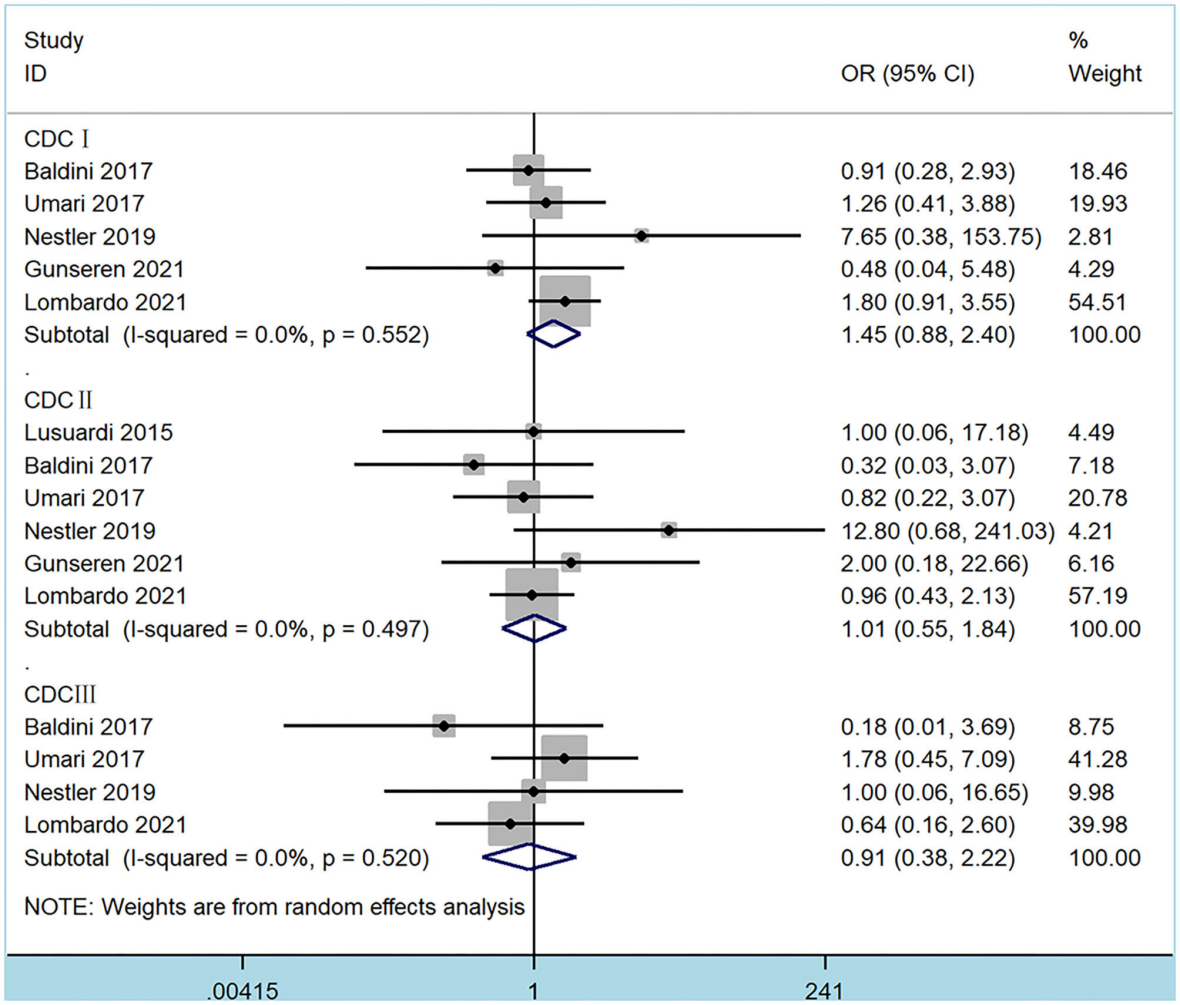
Forest plots of Clavien-Dindo classification of complications.

The pooled results demonstrated that MISP and EEP techniques had similar overall complications (OR 1.10, 95% CI 0.76 to 1.59, *p* = 0.62) and blood transfusions (OR 1.83, 95% CI 0.66 to 5.08, *p* = 0.24) (Table 3). Moreover, complications of CDC grade I (OR 1.45, 95% CI 0.88 to 2.40, *p* = 0.14), grade II (OR 1.01, 95% CI 0.55 to 1.84, *p* = 0.98) and grade III (OR 0.91, 95% CI 0.38 to 2.22, *p* = 0.84) were analyzed from 6 studies (Figure 4). No significant differences were found between MISP and EEP (all *p* > 0.05). Statistical heterogeneity between these studies was not significant (*p* > 0.10).

### Subgroup Analysis

Due to the limited number of studies available for meta-analysis, we only performed subgroup analyses for perioperative outcomes and complications and stratified by minimally invasive approaches (Table 3). For subgroup with LSP, the pooled results indicated that LSP was associated with longer operative time (*p* = 0.009), catheterization time (*p* = 0.0001) and LOS (*p* = 0.0008) compared with EEP. However, there were no significant differences in Hb decrease, resection weight, overall complications, and blood transfusions between the groups (all *p* ≥ 0.05). In terms of the RASP subgroup, patients undergoing RASP had higher Hb decrease (*p* = 0.0007), longer catheterization time (*p* = 0.04), longer LOS (*p* = 0.008) and higher blood transfusions (*p* = 0.004) compared to the EEP group. However, no differences were observed regarding operative time, resection weight, and overall complications between the groups (all *p* ≥ 0.05).

### Sensitivity Analysis

Due to the limited number of articles included in the study, we performed a sensitivity analysis of perioperative outcomes and complications. No significant change in the combined effect was observed by eliminating each study one by one, which demonstrates the stability of our results (Supplementary Material 2).

### Publication Bias

**Figure 5:**
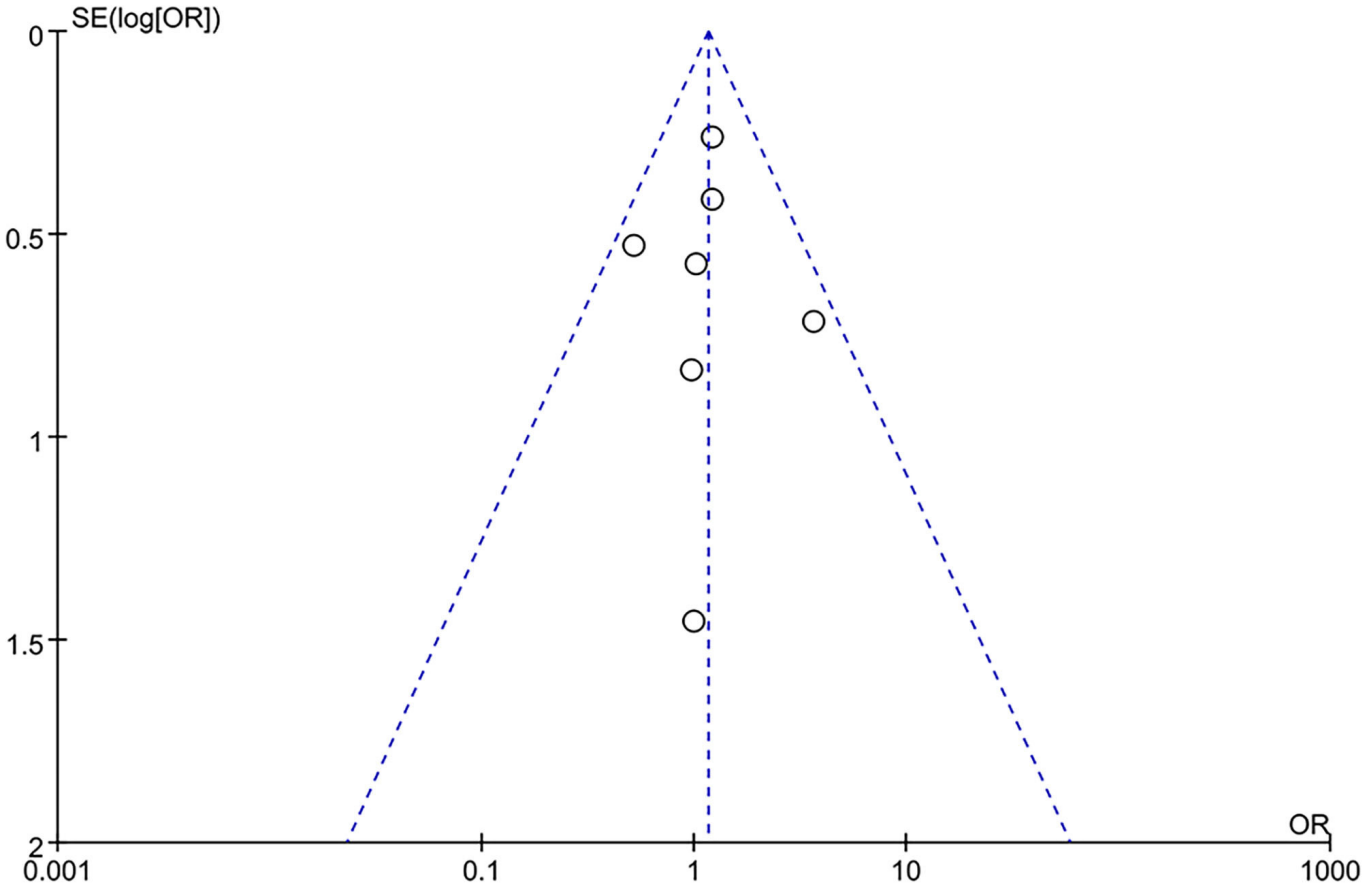
Funnel plot of publication bias.

Publication bias analysis was performed based on overall complications index, no significant publication bias was observed in the analysis with a funnel plot (Figure 5) and in Begg's test (*p* > 0.1).

## Discussion

Over the past several decades, OSP has been the surgical method of choice for treating large BPH, and it may disappear as minimally invasive methods continue to advance (18). EEP and MISP are currently the preferred alternative procedures, but the superiority of these two approaches remains controversial. A previous meta-analysis of comparatives studies by Abi (20) aimed at comparing MISP with laser enucleation revealed that the two methods had similar postoperative IPSS (*p* = 0.23) and major complications (*p* = 0.41). They also concluded that laser enucleation had a shorter operative time (*p* = 0.006), LOS (*p* < 0.001) and catheterization time (*p* < 0.001) than MISP. One problem with the Abi study is that although five studies were included for analysis, only two or three were available for comparison of postoperative functional outcomes. Therefore, with the publication of new studies related to this topic, further evaluation of the efficacy and safety of MISP and EEP is necessary.

We conducted a meta-analysis of clinical studies comparing MISP and EEP in terms of short-term efficacy, perioperative outcomes, and complications. According to the review criteria, 8 comparative trials involving 1,504 patients with large BPH were determined. The quantitative analysis demonstrated that the two surgical procedures had similar 3- and 6-month IPSS, 3- and 6-month Qmax, 3-month PVR, and 3-month QoL. Our findings are broadly consistent with previously published literature. This suggests that even though there are some differences between the different surgical procedures, they appear to be equally effective for treatment of large BPH (19). Furthermore, it should be noted that we also observed that EEP had a superior 6-month PVR (only two studies involved), but this conclusion needs to be confirmed in high-quality randomized controlled trials (RCTs) with longer follow-up.

In this meta-analysis, we found that EEP might have superior perioperative outcomes. Compared with the MISP group, the EEP group had a shorter operation time, less Hb decrease, shorter catheterization time, lower LOS, and comparable resection weight. Our results are consistent with the existing evidence comparing the two surgical methods (12, 15, 19). Interestingly, subgroup analysis revealed that when the two minimally invasive techniques were separately compared with endoscopic surgery, the operative time was higher for LSP (*p* = 0.009) and comparable for RASP (*p* = 0.08). Nevertheless, there was significant heterogeneity in these studies. Baldini et al. (13) and Fuschi et al. (17) reported that there was no difference in operative time between LSP and endoscopic procedure. In contrast, Gunseren et al. (18) observed that LSP was associated with higher operative time. They claimed that closing bladder incisions could lead to a partial increase in operative time, even in the hands of surgeons with laparoscopic experience. Further correlation line demonstrated that the operative time advantage of holmium laser enucleation of the prostate (HoLEP) might disappear in huge prostates. For comparison of RASP and endoscopic procedure, Zhang et al. (15) found that RASP had a higher operative time, while a prospective randomized study by Fuschi et al. (17) showed no difference in operative time between the two procedures. On the side, Fuschi et al. (17) also reported that RASP had comparable operative time to LSP.

The technical proficiency of the surgeon appears to be a factor affecting the duration of operation (8). Johnson et al. (21) found that the median operative time of RASP improved significantly as surgeons became more skilled (early cohort 160 mins vs. late cohort 134 mins). In addition, they also claimed that a physician with experience with robotic radical prostatectomy might need only 10–12 cases to become proficient with RASP. Nevertheless, surgeries that involve EEP, such as the HoLEP technique, still have a steep learning curve for even experienced surgeons (22). Brunckhorst et al. (23) reported that HoLEP required 40–60 cases to achieve a proficient efficiency platform. In consequence, the “learning curve” factor might be more favorable to RASP rather than HoLEP, but it still needs to be validated in high-quality, multicenter RCTs.

We found that the EEP had a lower Hb decrease compared to MISP. In fact, both procedures rely on direct application of energy to achieve intraoperative hemostasis, but the endoscopic method can control the bleeding rapidly during the enucleation and thus has a better hemostasis effect (15). In most cases, fewer bleeding may be associated with surgical parameters such as shorter catheterization time and lower LOS, so the EEP group might have shorter catheterization time and LOS (24). However, there were significant differences in the results of different studies. Lusuardi et al. (12) reported a lower Hb decrease in endoscopic surgery, whereas Umari et al. (14) and Lombardo et al. (19) did not observe such a significant difference. In addition, higher hemoglobin reduction did not translate into higher blood transfusions. We observed similar blood transfusions between the groups (7.4 vs. 3.1%, *p* = 0.24). Interestingly, the RASP subgroup had a higher rate of transfusion (*P* = 0.004). However, the transfusion rate may not be a good indicator for evaluating the outcome of minimally invasive surgery as they vary from among hospitals, according to patient comorbidities and surgeon preferences (25).

The present study also expanded the pooled evidence to show that the MISP group had comparable overall complications (*p* = 0.62) compared to the EEP group. Our subgroup analysis also indicated that there was no difference in overall complications between minimally invasive techniques (LSP and RASP) and endoscopic procedures. These results are consistent with the available evidence. In most studies, the incidence of related complications was low and appeared to be evenly distributed in the MISP and EEP groups. Overall, both MISP and EEP are safe surgical procedures for the treatment of large BPH, and no clear conclusions can be drawn in favor of endoscopic therapy.

Another major constraint on the widespread use of surgical techniques is cost. Juaneda et al. (26) observed an average savings of €1835 for HoLEP compared to LSP (€2871 vs. €4706), and proposed that the reduced cost of the HoLEP was associated with shorter LOS. Unfortunately, no studies have reported a cost comparison between RASP and HoLEP, but in the comparison between RASP and OSP, Matei et al. (27) found that the average cost of RASP was €3940. Besides, Sotelo et al. (28) noted that the mean cost of the RASP was $1627 more expensive than LSP. These results suggest that endoscopic procedures appear to be cheaper than MISP. However, cost-benefit analysis is extremely complex and is influenced by many factors, such as hospital costs, complications, and reimbursement issues, which vary widely between countries and healthcare systems (25). Therefore, carefully designed studies are needed before definitive conclusions can be drawn.

Compared with the study of Abi Chebel et al. (20), the present meta-analysis has the advantage of including three recent comparative trials and then analyzing more parameters related to the effectiveness and safety of these two methods. In addition, the previous study only provided 3-month IPSS data, whereas our study expanded the sample size and further evaluated functional outcomes at 6-month. Another strength of the current study was our subgroup analyses, which for the first time systematically compared RASP with EEP outcomes.

Limitations should be noted before interpreting our findings. First, this study was unable to analyze the learning curves and costs of the two processes because of the lack of data. Second, significant heterogeneity was observed in perioperative outcomes and complications, which may limit the strength of our evidence. For this, we conducted a subgroup analysis of each outcome indicator based on the minimally invasive approach, hoping that this has increased some robustness to our analysis and interpretation. Third, the follow-up time was mostly less than 6 months. Therefore, our results can only compare the short-term efficacy and safety of MISP and EEP. Finally, due to the inconsistent follow-up time in this study, there may be deviations in the reports of complications.

## Conclusion

The current study suggested that the short-term efficacy and safety were comparable between MISP and EEP procedures for large prostates. However, EEP may have a more favorable perioperative profile with shortened operative time, reduced Hb decrease, shortened reduced catheterization time, and reduced LOS. Based on these results, both surgical procedures could be offered to patients, which should understand the advantages and disadvantages of each method to make a suitable decision.

## Data Availability Statement

The original contributions presented in the study are included in the article/Supplementary Material, further inquiries can be directed to the Corresponding Author/s.

## Author Contributions

QW is responsible for manuscript conceptualization. JL, DC, and CM are responsible for manuscript writing and data analysis. LP and ZX are responsible for data analysis. YL and QW are responsible for manuscript editing. All authors contributed to the article and approved the submitted version.

## Funding

This work was supported by the National Natural Science Foundation of China (Grant Number 82000721), Post-Doctor Research Project, West China Hospital, Sichuan University (Grant Number 2019HXBH089), Health commission of Sichuan province (Grant Number 20PJ036), and Sichuan Province Science and Technology Planning Project (Grant Number 2020YJ0054 and 2020YFS0320).

## Conflict of Interest

The authors declare that the research was conducted in the absence of any commercial or financial relationships that could be construed as a potential conflict of interest.

## Publisher's Note

All claims expressed in this article are solely those of the authors and do not necessarily represent those of their affiliated organizations, or those of the publisher, the editors and the reviewers. Any product that may be evaluated in this article, or claim that may be made by its manufacturer, is not guaranteed or endorsed by the publisher.
