# Supplementary material for: Comparison of the Efficacy and Safety of Minimally Invasive Simple Prostatectomy and Endoscopic Enucleation of Prostate for Large Benign Prostatic Hyperplasia
Source: Front Med (Lausanne). 2021 Nov 5;8:773257. doi: 10.3389/fmed.2021.773257 (PMC8602691; doi:10.3389/fmed.2021.773257)
Supplement: Supplementary file 1 [file Table_1.docx]

| **Supplementary File 1.** Quality evaluation of the eligible studies with Newcastle–Ottawa scale. | | | | | | | | | |
| --- | --- | --- | --- | --- | --- | --- | --- | --- | --- |
| Study | Selection | | | | Comparability | | Outcome | | |
|  | Representativeness | Selection of  non-exposed | Ascertainment  of exposure | Outcome not present at start | Comparability on most important factors | Comparability on other risk factors | Assessment of outcome | Long enough follow-up (median≥1 year) | Adequacy  (completeness) of follow-up |
| Lusuardi et al. | * | * | * | * | * | - | * | - | * |
| Baldini et al. | * | * | * | * | * | * | * | - | * |
| Umari et al. | * | * | * | * | * | * | * | - | * |
| Zhang et al. | * | * | * | * | * | - | * | - | * |
| Nestler et al. | * | * | * | * | * | - | * | * | * |
| Fuschi et al. | * | * | * | * | * | * | * | * | * |
| Gunseren et al. | * | * | * | * | * | - | * | - | * |
| Lombardo et al. | * | * | * | * | - | * | * | * | * |
| *indicates criterion met; - indicates significant of criterion not met. | | | | | | | | | |
