# Supplementary material for: Comparison of the Efficacy and Safety of Minimally Invasive Simple Prostatectomy and Endoscopic Enucleation of Prostate for Large Benign Prostatic Hyperplasia
Source: Front Med (Lausanne). 2021 Nov 5;8:773257. doi: 10.3389/fmed.2021.773257 (PMC8602691; doi:10.3389/fmed.2021.773257)
Supplement: Supplementary file 2 [file Table_2.docx]

**Supplementary File 2**. Sensitivity analysis of perioperative outcomes and complications.


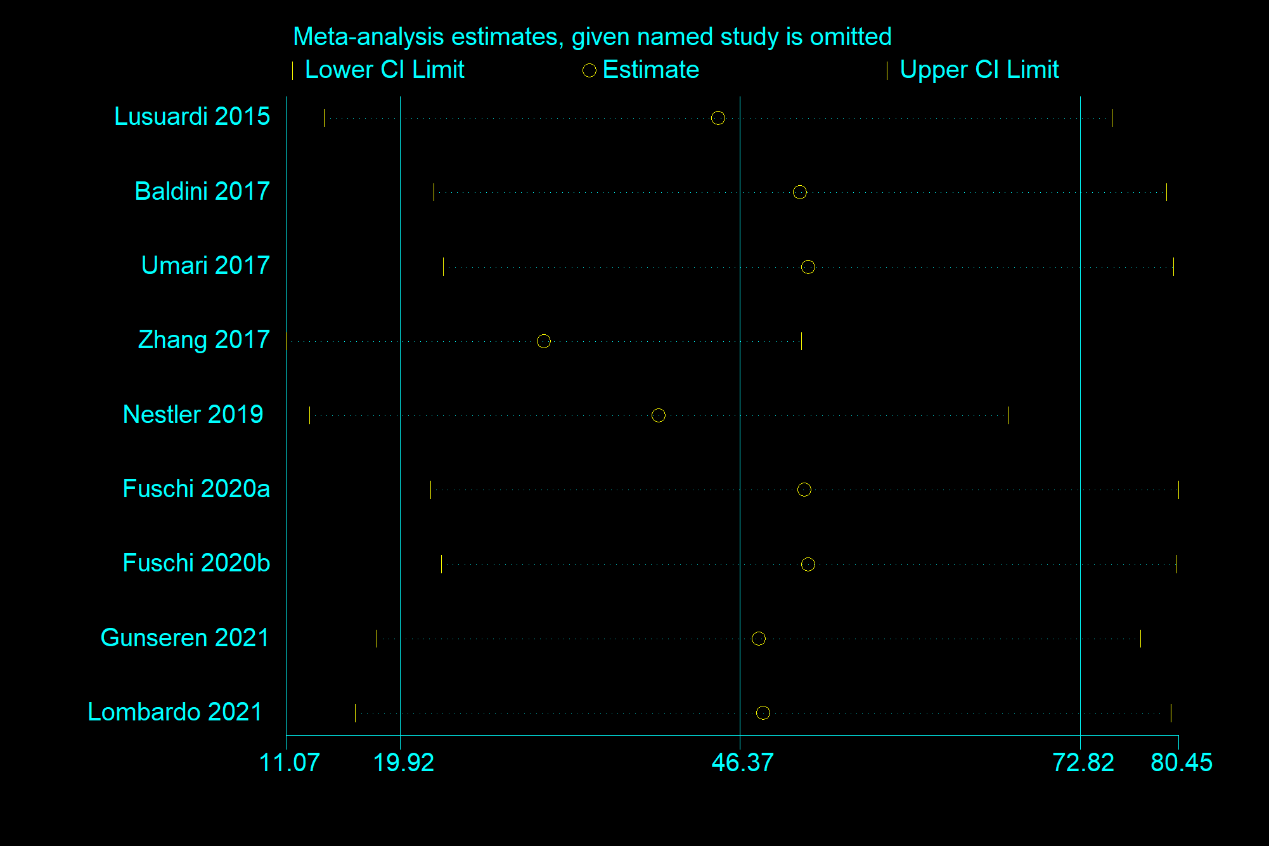


**A: Sensitivity analysis of operating time**


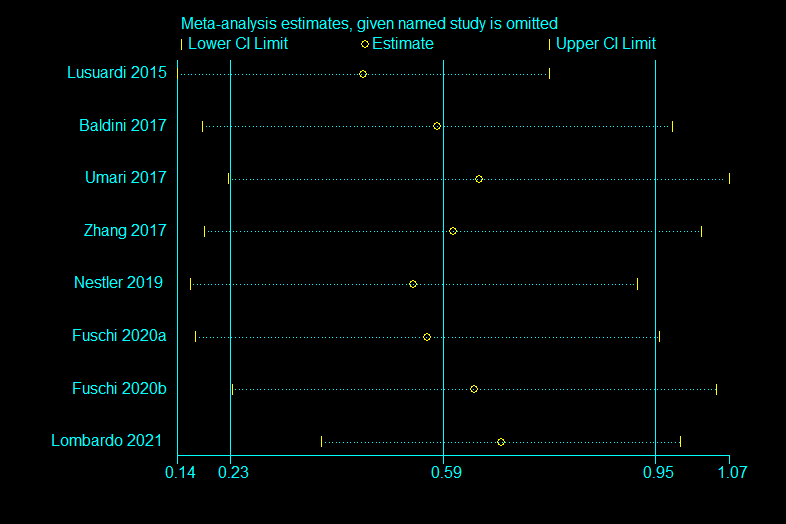


**B: Sensitivity analysis of hemoglobin decrease**


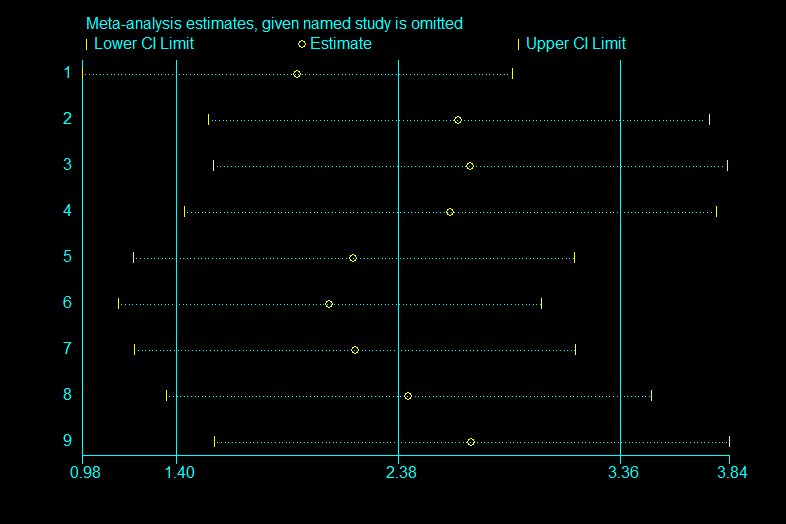


**C: Sensitivity analysis of length of stay**


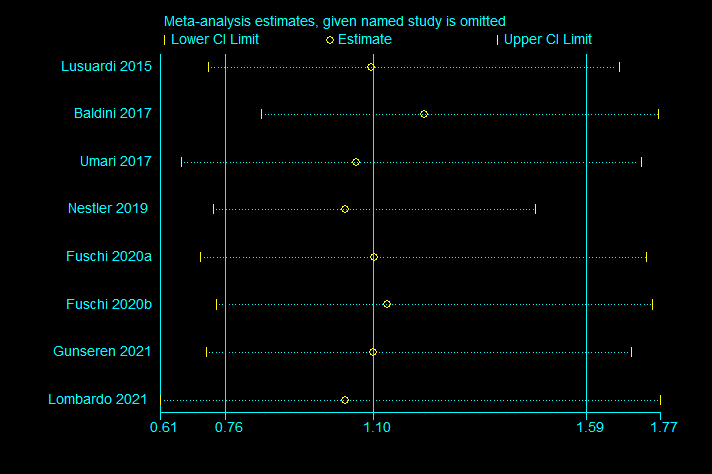


**D: Sensitivity analysis of complications**

**
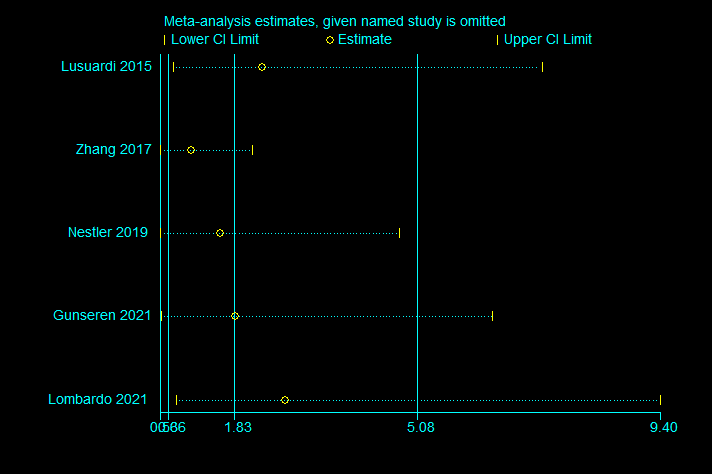
**

**E: Sensitivity analysis of blood transfusions**
